# Effect of Chronic Corticosterone Treatment on Depression-Like Behavior and Sociability in Female and Male C57BL/6N Mice

**DOI:** 10.3390/cells8091018

**Published:** 2019-09-01

**Authors:** Stefanie Berger, Sarah Gureczny, Sonali N. Reisinger, Orsolya Horvath, Daniela D. Pollak

**Affiliations:** Department of Neurophysiology and Neuropharmacology, Center for Physiology and Pharmacology, Medical University of Vienna, Schwarzspanierstrasse 17, 1090 Vienna, Austria

**Keywords:** depression, corticosterone, mouse, behavior, sex-effect

## Abstract

Depression is a very common psychiatric disorder affecting approximately 300 million people worldwide with the prevalence being twice as high in women as in men. Despite intense research efforts in recent decades, the neurobiological basis underlying depression remains incompletely understood. However, the exposure to chronic stress is widely accepted to constitute a precipitating factor for the development of this mental disorder. Several animal models for the investigation of the pathogenetic link between chronic stress and depression exist and have yielded important insights. The present study aimed at comparing two published protocols for the induction of depression-like behavior in mice based on chronic oral glucocorticoid application. Given the gender distribution in the prevalence of depression, the second goal of this study was to reveal possible differences in the behavioral responses of female and male mice to corticosterone (CORT) treatment. CORT treatment was found to modulate depression-like behavior in selected behavioral paradigms in a sex- and protocol-specific manner. These data are of relevance for the experimental design and interpretation of future studies in the field and further highlight the relevance of “sex as biological variable” to be considered an important parameter for experimental planning and interpretation of results.

## 1. Introduction

Major depressive disorder (MDD) is a mental disorder affecting more than 300 million people worldwide (4.4% of the world’s population) and the number is increasing continually. Apart from the socioeconomic relevance, with MDD accounting for the largest part of work-related disabilities and the significant treatment costs associated with the therapeutic management of affected patients, MDD is also a significant contributor to the annual 800,000 suicide cases [1]. Symptomatic characteristics of MDD include depressed (low) mood, loss of interest and pleasure, significant weight change, social withdrawal, feeling worthless and guilty, sleep disturbances, and recurrent thoughts of suicide [2]. 

Despite the fact that a range of endogenous and exogenous factors are known to be involved in the pathogenesis of depressive disorders, the underlying pathophysiological mechanisms remain poorly understood. The limited understanding of the neural underpinnings of depression, which also hampers the development of importantly needed alternative treatment approaches, is in part related to the lack of availability of adequate R&D test systems. Several animal models for depression are available [3]. However, most have been designed and validated in male subjects only, despite the fact that the prevalence of MDD is twice as high in women than in men [4]. Although this background suggests the female organism as primary target for drug development, surprisingly few protocols have been tested in female and male subjects. Moreover, the paradigms traditionally used to evaluate the behavioral endpoints of these models, have been majorly developed in male animals and are therefore tailored to more readily detect changes in male-defined behavioral patterns [5]. The most commonly assessed behavioral displays considered of relevance for mood disorders are behavioral despair, anhedonia, and depression-related anxiety [6,7,8,9]. Albeit, the fact that social withdrawal is a major symptom of MDD, sociability and a lack thereof is often not evaluated in animal models of depression, although it can be readily detected in the social interaction task. Indeed, while the more commonly used paradigms, such as the Forced Swim test (FST), the Tail Suspension Test (TST), or the Novelty Suppressed Feeding test (NSF) are based upon the examination of a behavioral response in a given stressful situation, social interaction allows for the characterization of a spontaneously displayed behavior. 

Exposure to stressful life events like unemployment, abuse, physical trauma, and financial and relationship problems [10] is associated with an enhanced risk for the development of several psychiatric disorders, including MDD. Persistent stress induces structural and functional neural alterations in brain regions forming part of the neural circuitry underlying depression [11] and alterations in the endogenous stress response system, centered around the activity of the hypothalamic-pituitary-adrenal (HPA) axis, have been consistently reported in depressed patients [12,13]. Accordingly, several stress-based animal models exist, including chronic mild stress paradigms, inescapable stress, and social defeat stress. As exposure to any type of stressor ultimately activates the HPA axis, leading to the secretion of corticosterone (CORT), long-term systemic CORT administration procedures were developed as additional models [14,15]. CORT-treated animals have been reported to present with several depression-like behavioral features, including augmented behavioral despair in the FST and anhedonia in Sucrose Preference Test (SPT) [16,17,18], as well as an increased latency to approach the food in the NSF, interpreted as hyponeophagia [19]. 

However, a thorough characterization of the response of female rodents to chronic CORT exposure is not available to date and the consequences of chronic CORT treatment for sociability have remained unexplored so far. Here we employed and compared two different chronic CORT protocols in female and male C57Bl/6N mice with regard to behaviors in three of the most commonly used depression-relevant tasks (i.e., FST, SPT, NSF) and additionally evaluated the impact of long-term CORT treatment on sociability in the Social Interaction test (SI).

## 2. Materials and Methods

### 2.1. Animals

C57BL/6N mice were used for all experiments. Animals were purchased from Charles River (Sulzfeld, Germany) at 7–8 weeks of age and were allowed to habituate for at least one week upon arrival to the local animal facility before initiation of experiments. All animals were single-housed under standard conditions in a colony room with a temperature of 22 ± 1 °C, a 12:12 h light/dark cycle and food and water available *ad libitum* unless stated otherwise. The light intensity amounted to about 10–20 lux inside the home cages. All experiments concerning mice were carried out respecting the ARRIVE guidelines and the U.K. Animals (Scientific Procedures Act, 1986 and associated guidelines, EU Directive 2010/63/EU for animal experiments) and were approved by the national ethical committee on animal care and use (BMBWF-66.009/0094-V/3b/2018- Bundesministerium für Wissenschaft und Forschung).

### 2.2. CORT Treatment

Two different protocols were used for oral chronic CORT application and adapted to the same concentration of 35 µg/mL:

1. The first protocol (“CORT1”) was adapted from David et al. [19]. 35 mg of corticosterone powder (SIGMA, cat. No. 27840) were dissolved in 10 mL 45% (2-Hydroxypropyl)-β-cyclodextrin (SIGMA, cat. No. 332593) for a final concentration of 35 µg/mL in 1 L of tap water (“CORT1”). The control group in this paradigm received 0.45% β-cyclodextrin in tap water (“Vehicle1”).

2. The second protocol (”CORT2”) was adapted from Gourley & Taylor [16]. The appropriate amount of CORT hemisuccinate was corrected for the added molecular weight of the salt multiplying the desired dose of 35 mg by a factor of 1.289. Therefore, 45.11 mg of corticosterone hemisuccinate (STERALOIDS, INC, cat. No. Q1562-000) were added to 1 L of tap water (CORT2). The pH of the solution was adjusted to 7.2 using hydrochloric acid. Control mice received tap water with the same pH adjustment (“Vehicle2”). 

Solutions were prepared freshly every 72 h in all instances and kept light protected in specufuc drinking bottles.

**Figure 1:**
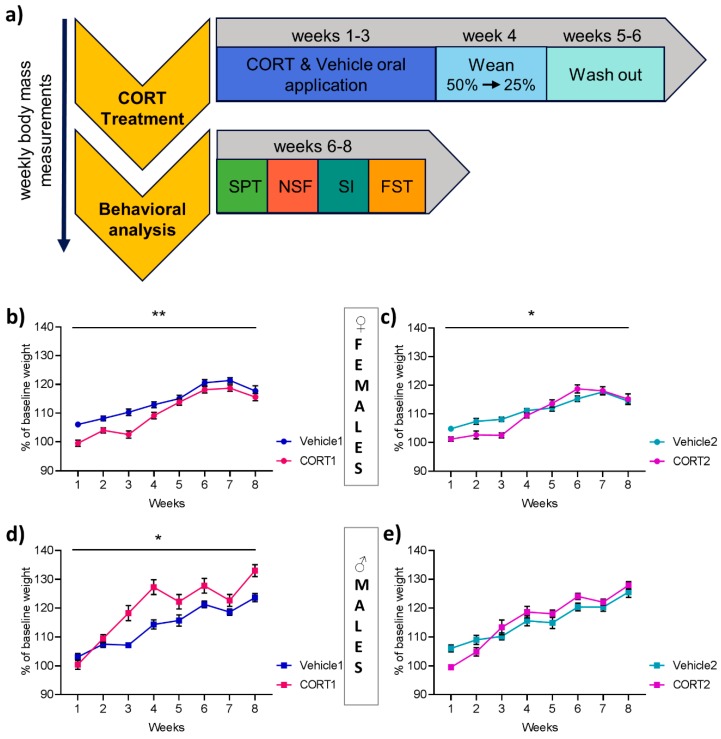
Study design and bodyweight changes resulting from CORT treatment. (**a**) Study design with experimental timeline. Weekly bodyweight measurement in (**b**–**c**) female mice and (**d**–**e**) male mice of each CORT and Vehicle group (*n* = 15 per group). All values are presented as mean ± SEM. Statistical significances displayed are results of repeated measure ANOVA. * *p* < 0.05; SPT: Sucrose preference test; NSF: Novelty suppressed feeding test; SI: Social interaction test; FST: Forced swim test.

CORT treatment lasted 3 weeks, followed by a weaning phase during which mice received a solution at 50% and then 25% of the full dose for 3 days, respectively, with a subsequent washout period of 2 weeks to allow for the investigation of long-lasting effects of chronic CORT as originally described [16] (Figure 1a).

A total of 120 mice (60 males and 60 females) were randomly assigned to one of the 4 treatment/ control groups (*n* = 15/group/sex) and the bodyweight of each animal was recorded once per week. Male and female animals were analyzed separately each in two consecutive cohorts. In an effort to reduce the impact of potentially occurring subtle changes in the housing and testing environments which may impact on the behavioral performance, all results were analyzed in relative terms such that data derived from each CORT groups were normalized to their respective controls which were tested in parallel. 

### 2.3. Behavioral Analysis

#### 2.3.1. Sucrose Preference Test (SPT)

The SPT assessing anhedonic behavior was adapted from a standard protocol [20,21]. Briefly, after a 48 h habituation phase to the 2% sucrose (Sigma Aldrich, Vienna, Austria) solution, animals were food and water restricted for 18 h before the actual testing. During the 3 h testing period mice given the choice to drink from either of two identical bottles, one filled with 2% sucrose solution, and the other with regular tap water. The weight of the bottles before and after testing was monitored and used for the calculation of the percentage of sucrose preference in reference to the total liquid consumption.

#### 2.3.2. Novelty Suppressed Feeding (NSF)

The NSF was conducted according to a previous study [22,23]. Briefly, mice were weighed and food-restricted 24 h prior to behavioral testing. A single food pellet was placed into the center of a brightly lit (700 lux) arena (30 × 50 cm) filled with wood-chip bedding material. The latency to feed on the pellet was recorded (15 min maximum time) for each mouse and used as a parameter for the indication of depression-related anxiety.

#### 2.3.3. Social Interaction (SI)

SI was conducted in a rectangular three-chambered sociability cage (Noldus, Wageningen, Netherlands), in which one wired cage for the unfamiliar mice or the novel object was placed in the left and the right side compartment. The test mouse was allowed a 15 min habituation period after which an unfamiliar, age- and sex-matched C57BL/6N mouse, labeled “stranger“, was placed in a wire cage on one side of the arena, whilst a “dummy“ mouse (black Lego^®^ blocks) was placed in the other compartment. The trial was video-recorded for 10 min and analyzed using Ethovision12XT^®^ (Noldus, Wageningen, The Netherlands) by evaluating the time spent in the compartment containing the stranger mouse in comparison to the amount of time spent in the compartment of the object.

#### 2.3.4. Forced Swim Test (FST)

Behavioral despair in the FST was evaluated according to a standard protocol [24,25]. Briefly, mice were placed in a transparent beaker filled with water (22–24 °C) for 6 min. The movements of the mice were videotaped and automatically recorded by a tracking software (VIDEOTRACK (PORSOLT), Viewpoint, Champagne au mont d’Or, France). The percentage of time spent immobile during the last 4 minutes of the test was calculated and used as indicator for behavioral despair.

### 2.4. Statistical Analysis

Results of CORT treated mice were normalized against the mean of their respective control group. All data were tested for normality using Kolmogorov-Smirnov test prior to further statistical evaluation. All behavioral experiments, except SI, were analyzed using unpaired two-tailed Student’s t-test or corrected with Welch’s t-test, where appropriate. Bodyweight measurements and SI were evaluated using repeated measure one-way ANOVA with posthoc pairwise comparison. 

Statistical outliers (values outside of the interval: mean ± 2 standard deviations, which covers >95% of a normal distribution) were excluded from further analysis. 

All statistical analyses were performed using SPSS software, for windows, Version 24 (IBM Corporation, Chicago, IL, USA) and Graphpad Prism, Version 7 (San Diego, CA, USA). A summary of all statistical results is provided in Table S1.

## 3. Results

The present study compared two published protocols of chronic oral glucocorticoid application, previously used for the induction of depression-like behavior in mice [16,19]. Behavioral analysis (SPT, NSF, SI and FST (in order from the least stressful to the most stressful test, with the observation of a minimal 24 h inter-test interval)) was conducted at the end of the entire treatment period which consisted of 3 weeks of full CORT application, followed by 6 days of weaning and a washout period of 2 weeks (Figure 1a). The drinking volume during the treatment phase was measured and no significant difference was observed between all female groups and males of Vehicle1 vs. CORT1. However, males of CORT2 showed significantly higher drinking volume (t(17.46) = 2.49, *p* < 0.05, Supplementary Figure S2a–d) compared to their respective control group (Vehicle2).

### 3.1. Effect of CORT Treatment on Bodyweight Increase

Body weight for each animal was recorded throughout the experiment and showed the expected overall increase over time in female and male animals. Statistical analysis revealed a significant main effect of treatment between CORT and controls in females of both CORT groups (F(1,12) = 9.20, *p* ≤ 0.01, Figure 1b and F(1,13) = 5.77, *p* < 0.05, Figure 1c). In CORT1 vs. Vehicle1 an interaction between time and treatment was also revealed (F(1,12) = 4.16, *p* ≤ 0.01), where posthoc multiple comparisons showed differences in weeks 1–4 (*p* < 0.05). However in CORT2 vs. Vehicle2 only a trend, short of statistical significance, was observed (F(1,12) = 2.11, *p* = 0.075). In males a difference between CORT and controls in Vehicle1 vs. CORT1 was revealed with a main effect of treatment (F(1,17) = 7.76, *p* < 0.05, Figure 1d) and a significant interaction between time and treatment (F(1,17) = 3.61, *p* ≤ 0.01), where posthoc pairwise comparison demonstrated differences in week 3–4 and 6–8 (*p* < 0.05). For male Vehicle2 and CORT2 no treatment effect but a significant interaction between time and treatment was observed (F(1,17) = 4.22, *p* < 0.01), with significant differences in week 1 (*p* < 0.05) as resulting from pairwise comparisons (Figure 1e).

In order to evaluate the effects of CORT treatment on depression-like behavior the performance of all mice in the SPT, NSF, SI, and FST was determined.

### 3.2. No Impact of CORT Treatment on Sucrose Preference

**Figure 2:**
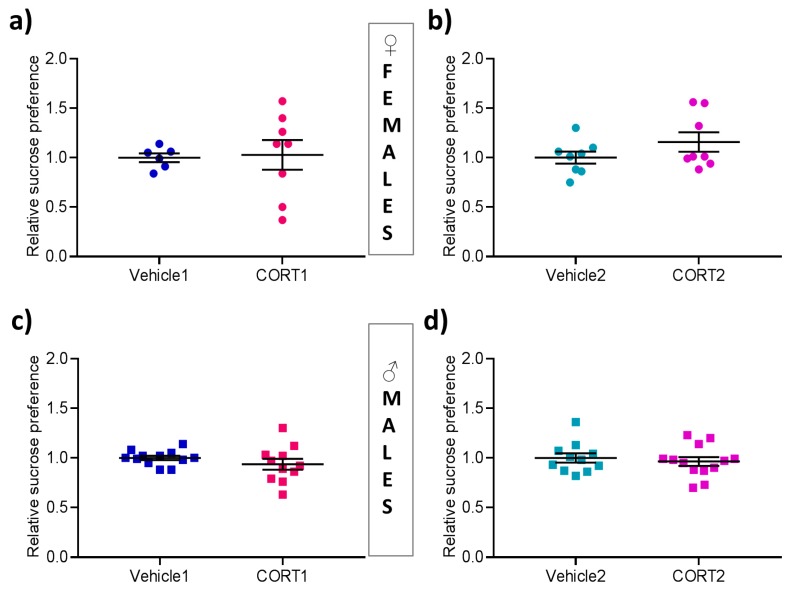
Results of Sucrose Preference Test in CORT mice. Evaluation of the sucrose preference test showing relative % of sucrose preference in (**a**–**b**) female (**c**–**d**) male mice. All values are presented as mean ± SEM (*n* = 6–13 per group). Statistical significances indicate results of Student‘s t-test. * *p* < 0.05.

In the SPT the percentage of sucrose preference over water was calculated as a proxy measure of hedonic behavior. No significant difference between Vehicle and CORT groups for both treatment protocols was observed in female and male mice (Figure 2a–d).

### 3.3. Altered Hyponeophagia in CORT Mice

**Figure 3:**
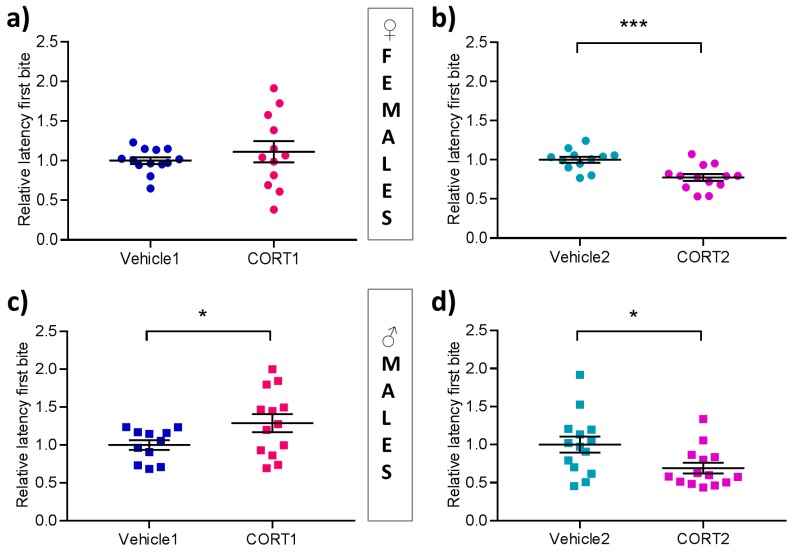
Novelty Suppressed Feeding test in CORT treated and control mice. Behavioral outcome of the novelty suppressed feeding test showing relative latencies until the first bite in (**a–b**) female (**c–d**) male mice of each CORT and Vehicle group. All values are presented as mean ± SEM (*n* = 11–14 per group). Statistical significances are results of Student‘s t-test. * *p* < 0.05, *** *p* < 0.001.

In the Novelty Suppressed Feeding test, evaluation of depression-related anxiety-like behavior was determined by the latency until the first bite. Female mice of Vehicle1 vs. CORT1 group showed no significant differences in the latency (Figure 3a), however in females of Vehicle2 vs. CORT2 a significant difference was detected (t(23) = 3.88, *p* < 0.001, Figure 3b), with CORT2 treated female mice exhibiting a shorter mean latency than their vehicle control group. Male mice of the CORT1 group showed a significant decrease in latency with respect to the Vehicle1 control (t(18.12) = 2.13, *p* < 0.05, Figure 3c), while a significant increased latency in CORT2 compared to control animals was observed (t(26) = 2.44, *p* < 0.05, Figure 3d).

In order to control for possible unspecific effects of CORT treatment on bodyweight and food drive, the percentage of bodyweight loss during the food deprivation period prior to the NSF was determined and home cage food consumption was measured. For the latter, mice are placed back into their home cage immediately after the NSF and a pre-weighed pellet of food is presented for 5 min. No significant differences in home cage food consumption between treatment groups were observed for both sexes (Supplementary Figure S1a–d). Body weight loss was measured by weighing the mice before the 24 h food restriction and immediately before placing the mice into the novel arena of the NSF. No significant differences were observed for any of the experimental groups (Supplementary Figure S1e–h).

### 3.4. Impaired Social Interaction in CORT Mice

**Figure 4:**
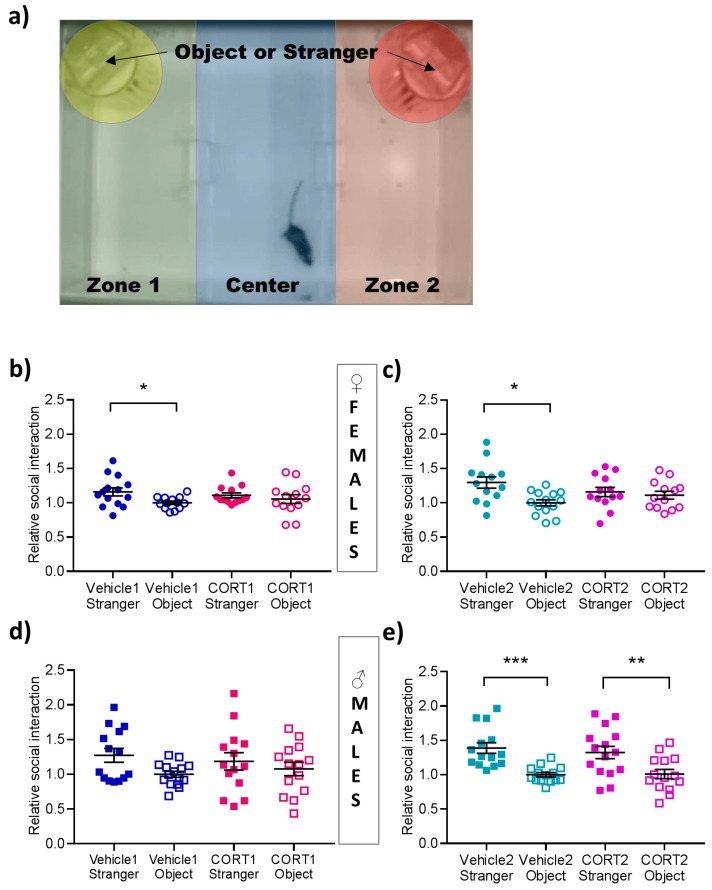
Social Interaction test in CORT mice. (**a**) Experimental design and behavioral results of social interaction test in (**b**–**c**) female and (**d**–**e**) male CORT treated and Vehicle control mice. All values are presented as mean ± SEM (*n* = 13–15 per group). Statistical significances displayed results of posthoc pairwise comparison after repeated measure ANOVA. * *p* < 0.05, ** *p* < 0.01, *** *p* < 0.001.

The social interaction test evaluates the preference of a mouse to interact with a stranger mouse over an inanimate object (dummy mouse) (Figure 4a). A main effect of social preference for the stranger mouse over the object was shown in females of CORT1 paradigm (F(1,24) = 4.41, *p* < 0.05), in females of CORT2 paradigm (F(1,24) = 4.70, *p* < 0.05) and in males of CORT2 paradigm (F(1,26) = 26.37, *p* < 0.0001). However, this preference was not present in male mice of CORT1 paradigm (Figure 4d). Posthoc pairwise comparison results showed that intact sociability was detectable in both female vehicles groups (*p* < 0.05, Figure 4b,c); however, social preference was absent in both CORT groups of female mice. This absence was not reflected in CORT2 of male animals (*p* < 0.01) showing a social preference comparable to their respective Vehicle group (*p* < 0.0001, Figure 4e).

### 3.5. CORT2 Females Present with Increased Behavioral Despair

**Figure 5:**
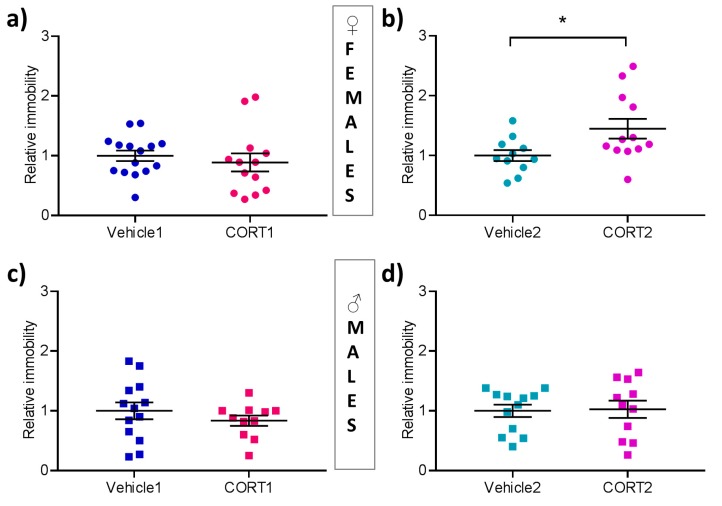
Behavioral despair in the Forced Swim Test in CORT mice. Behavioral analysis in the Forced Swim Test evaluating relative immobility time in (**a**–**b**) female (**c**–**d**) male CORT treated in and Vehicle control mice. All values are presented as mean ± SEM (*n* = 11–15 per group). Statistical significances are results of Student‘s t-test. * *p* < 0.05.

In the Forced Swim Test depression-related behavioral despair is assessed by the evaluation of the percentage of time animals spent immobile while being subjected to a beaker filled with water. Here, no group differences were observed in female mice for the CORT1 treatment (Figure 5a) while CORT2 female mice spent more time immobile (t(21) = 2.33, *p* < 0.05, Figure 5b) than Vehicle2 animals. No effect of either CORT treatment on immobility was detectable in male mice (Figure 5c–d).

## 4. Discussion

The present study firstly compared the effects of two different published protocols of chronic oral corticosterone treatment on depression-like behavior and sociability in female and male wildtype mice and revealed protocol and sex-specific behavioral phenotypes (see Table 1 for a summary of detected behavioral changes).

Both CORT treatment protocols have been initially published based upon experiments conducted in male mice, albeit other CORT treatment paradigms have also been applied to female rodents [26,27,28]. These studies have resulted in diverging conclusions regarding the sensitivity of female mice to develop alterations in emotional behavior upon chronic CORT application [27,28]. Here, we are relating our findings specifically to the information available for the protocols used in the present study [16,19].

However, several important procedural differences between the two protocols and with respect to the experiments described herein have to be considered for their potential relevance to the partly differing results. Importantly, as a central point of interest of the present study was to address the question of whether there are behavioral effects of the drug formulation vehicles used for the solubilization of CORT, we adapted the original protocols [16,19] in an effort to render the two paradigms comparable in terms of dose and timing. These modifications, including the incorporation of a 2 weeks washout period (originally part of the procedure described by Gourley et al. [16], but not by David et al. [19]) may account for some of the phenotypic discrepancies between the present and previous studies.

Additional diverging parameters are the mouse strains used (David et al. used C57BL/6Ntac while Gourley et al. carried out their work in C57BL/6 without providing information on the specific substrain employed [16,19] and we employed C57BL/6N mice for the present study) and the housing conditions (group-housing versus single-housing) are deemed particularly relevant. Here, we opted to single-house all experimental animals in order to exclude the effects of social hierarchy and related within-group phenotypic variability determined by social dominance ranks [29]. On the other hand, it has to be noted that single-housing might affect stress responsivity and coping abilities [30], albeit per se not sufficient to trigger an acute stress response [31]. Moreover, the age of the experimental animals at the onset of treatment (7–8 weeks herein and in David et al. [19] vs. at least 12 weeks of age as described by Gourley et al. [16]) is likely to constitute a factor determining the behavioral phenotype and differences between studies.

The previously published reports indicate increase in bodyweight in CORT treated in comparison to vehicle-treated animals for CORT1 [19] and no significant changes for CORT2 [16]. Here we replicate these finding in CORT1 (increase) and CORT2 (no change) mice and interestingly observe different effects in the female groups with both CORT treatment groups showed overall lower weight increase compared to their corresponding vehicle controls, suggesting distinct metabolic responses of female and male mice to chronic CORT exposure, which is in line with a recent report in female rats [26].

In order to evaluate behavioral phenotypes resulting from CORT treatment, we turned towards paradigms routinely employed to evaluate depression-like behaviors in mice. In addition to the traditionally used tests assessing anhedonia, depression-related anxiety and behavioral despair we also characterized sociability, which had not been previously assessed in the CORT chronic stress model.

In our experimental set-up, no differences between CORT treatment groups of male and female mice and their respective controls were observed for CORT1. Using the CORT2 protocol the treatment has previously been shown to reduce sucrose preference [16] in male mice, which was not observed herein in either female or male mice. In addition to the previously mentioned variables, also, the details of the SPT protocol applied could have been of relevance for the divergence in results.

In the NSF our results in the CORT1 group agree with the earlier description in male mice [19] with CORT1 treated males showing higher latency than animals of the Vehicle1 group, while no effect in female mice was observed. For the CORT2 groups, we found reduced latencies for both female and male mice which was unexpected considering that shorter latencies to bite in the NSF are typically related to a reduction in depression-related anxiety-like behavior. Gourley et al. [16] had not tested their animals in the NSF and the protocol and sex-differences are intriguing. Importantly, these observations again highlight the importance of including both sexes when evaluating behavioral endpoints in animal models of depression.

In the social interaction test where the time spent in the zone near a stranger mouse in a three-compartment arena is a reliable parameter indicating sociability [32], a decrease in time spent with the stranger mouse has been associated with depression-like behavior in other animal models [33] but not yet evaluated in response to chronic CORT treatment. Here, the expected intact sociability was revealed for most vehicle groups, while in female mice of both CORT paradigms social interaction was impaired. However, social preference was absent in Vehicle1 and CORT1 male mice, while CORT2 male mice showed sociability similar to the vehicle controls.

The FST, which was conducted last as it is considered to be most stressful [8,34], revealed no difference between CORT1 treated and Vehicle groups which agrees with the report by David et al. [16,19] in male mice. However, in the CORT2 groups we could not observe increased immobility scores in males, as previously described by Gourley et al. [16]. Female CORT2 mice did present with a significant increase in the time spent immobile in this test, suggesting an enhanced expression of despair in females or sex-specific differences in coping strategies [35] in our setup.

This study is the first to evaluate the behavioral outcomes of two different protocols of chronic oral CORT application in mice, based upon a comprehensive characterization of depression-like phenotypes in both sexes. Apart of slight discrepancies to previous literature, which may arise from methodological differences (protocol, circadian timing, devices) but also from fluctuations in housing (temperature, cage type, single versus group housing, food etc.) and strain varieties of the experimental animals used [36,37,38,39], the differences in the performance of female and male subjects are striking. Using a chronic mild stress paradigm in rats it has recently demonstrated that the increase in CORT from baseline levels is higher in female rats after stress exposure than in males [40], a finding which is paralleling observations of higher HPA-axis activity in response to stress in women than in men [41]. Hyperactivity of the HPA-axis, is a frequent finding in mood disorder patients [42,43]. At the molecular level, striking differences in the response to stress has been found between female and male mice as reflected in differential gene expression in the hippocampus, a brain region not known to present with prominent sex differences, but an abundant expression of GRs as well as sex hormone receptors [44]. A sex-dependent gene expression pattern may well also explain the differences in the behavioral phenotype of female and male mice in response to CORT treatment in the current study, a possibility that remains to be experimentally examined in future studies.

However, the present study complements existent literature to support the hypothesis that sexual dimorphism in the stress response system may contribute to the enhanced prevalence of depression among the female population.

In addition to emphasizing the importance of inclusion of sex as biological variable in biomedical research [45] and the well-known implications of this principle, our results also show that specific behavioral endpoints should be considered in experimental models of depression, depending on the sex of experimental animals. Notably, the effect of CORT treatment on sociability, which has not been previously described, appears as most stable among female subjects. These results are most relevant in light of the increasing evidence for a major role of social withdrawal in MDD as described above and against the background the sociability is not yet routinely assessed in animal model of depression.

In conclusion, we uncover sex-specific outcomes of chronic CORT treatment on behavioral phenotypes in C57BL/6N mice and suggest the evaluation of social interaction as an additional, ethologically relevant and methodologically sound behavioral endpoint in preclinical depression research.

## Figures and Tables

**Table 1:** Summary of behavioral phenotypes. Summary of behavioral phenotype referring to the depression- like behavior in the different treatment groups. Green arrows show increased depression- like behavior, red arrows indicate decreased depression- like behavior and blue lines present no effect according to the behavioral outcome of the respective test.

	CORT1		CORT2	
Behavioral Test	Females	Males	Females	Males
**SPT**	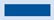	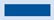	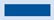	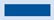
**NSF**	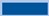	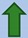	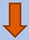	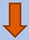
**SI**	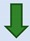	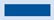	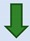	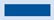
**FST**	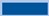	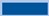	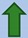	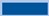

## Supplementary Materials

The following are available online at https://www.mdpi.com/2073-4409/8/9/1018/s1, Figure S1: Control experiments for Novelty Suppressed Feeding test, Figure S2. Drinking volume during treatment period, Table S1: Statistical summary of results.
