# Supplementary material for: Effect of Chronic Corticosterone Treatment on Depression-Like Behavior and Sociability in Female and Male C57BL/6N Mice
Source: Cells. 2019 Sep 1;8(9):1018. doi: 10.3390/cells8091018 (PMC6770122; doi:10.3390/cells8091018)
Supplement: Supplementary file 1 [file cells-08-01018-s001.zip › cells-560950-supplementary/SUPPL/Supplementary Table S1.pdf]

Supplementary Table S1. Statistical summary of results

Experiments	Sex	Comparison	Measurement	Statistical test	ANOVA factors	Statistics	Degrees of freedom	P-value	Figures
**Bodyweight**	female	Vehicle1 vs CORT1	% of baseline weight	Repeated measure ANOVA	Time	F = 73.82	df1 = 7 ; df2 = 12	**P<0.0001**	1b
					Treatment	F = 9.20	df1 = 1 ; df2 = 12	**0.0100**	
					Interaction	F = 4.16	df1 = 7 ; df2 = 12	**0.0010**	
		Vehicle2 vs CORT2			Time	F = 48.50	df1 = 5.05 ; df2 = 13	**P<0.0001**	1c
					Treatment	F = 5.77	df1 = 1 ; df2 = 13	**0.0320**	
					Interaction	F = 2.11	df1 = 5.05 ; df2 = 13	0.0750	
	male	Vehicle1 vs CORT1			Time	F = 68.92	df1 = 3.61 ; df2 = 17	**P<0.0001**	1d
					Treatment	F = 7.76	df1 = 1 ; df2 = 17	**0.0130**	
					Interaction	F = 5.26	df1 = 3.61 ; df2 = 17	**0.0010**	
		Vehicle2 vs CORT2			Time	F = 74.34	df1 = 3.56 ; df2 = 17	**P<0.0001**	1e
					Treatment	F = 1.49	df1 = 1 ; df2 = 17	0.239	
					Interaction	F = 4.22	df1 = 3.56 ; df2 = 17	**0.0060**	
**SPT**	female	Vehicle1 vs CORT1	Relative sucrose preference	Unpaired t-test		t = 0.19	df1 = 8.20	0.8566	2a
		Vehicle2 vs CORT2		Welch t-test		t = 1.37	df1 = 14.00	0.1923	2b
	male	Vehicle1 vs CORT1		Unpaired t-test		t = 1.07	df1 = 12.98	0.3032	2c
		Vehicle2 vs CORT2		Welch t-test		t = 0.55	df1 = 22.00	0.5876	2d

**NSF**	female	Vehicle1 vs CORT1	Relative latency to first bite	Welch t-test		t = 0.79	df1 = 13.23	0.4421	3a
		Vehicle2 vs CORT2		Unpaired t-test		t = 3.88	df1 = 23.00	**0.0008**	3b
	male	Vehicle1 vs CORT1		Welch t-test		t = 2.13	df1 = 18.12	**0.0476**	3c
		Vehicle2 vs CORT2		Unpaired t-test		t = 2.43	df1 = 26.00	**0.0220**	3d
**SI**	female	Vehicle1 vs CORT1	Relative social interaction	Repeated measure ANOVA	Social preference	F = 4.41	df1 = 1 ; df2 = 24	**0.0351**	4b
					Treatment	F = 0.02	df1 = 1 ; df2 = 24	0.8820	
					Interaction	F = 1.26	df1 = 1 ; df2 = 24	0.2720	
		Vehicle2 vs CORT2			Social preference	F = 4.70	df1 = 1 ; df2 = 24	**0.0400**	4c
					Treatment	F = 0.03	df1 = 1 ; df2 = 24	0.8720	
					Interaction	F = 2.51	df1 = 1 ; df2 = 24	0.1260	
	male	Vehicle1 vs CORT1			Social preference	F = 2.71	df1 = 1 ; df2 = 26	0.1120	4d
					Treatment	F = 0.03	df1 = 1 ; df2 = 26	0.8740	
					Interaction	F = 0.46	df1 = 1 ; df2 = 26	0.5060	
		Vehicle2 vs CORT2			Social preference	F = 26.37	df1 = 1 ; df2 = 26	**P<0.0001**	4e
					Treatment	F = 0.21	df1 = 1 ; df2 = 26	0.6500	
					Interaction	F = 0.47	df1 = 1 ; df2 = 26	0.4999	
**FST**	female	Vehicle1 vs CORT1	Relative immobility	Unpaired t-test		t = 0.66	df1 = 26.00	0.5125	5a
		Vehicle2 vs CORT2		Unpaired t-test		t = 2.33	df1 = 21.00	**0.0298**	5b

	male	Vehicle1 vs CORT1		Unpaired t-test		t = 0.96	df1 = 22.00	0.3493	5c
		Vehicle2 vs CORT2		Welch t-test		t = 1.53	df1 = 18.39	0.8798	5d
**Food consumption**	female	Vehicle1 vs CORT1	Relative food consumption	Unpaired t-test		t = 0.24	df1 = 24.00	0.8115	Suppl. 1a
		Vehicle2 vs CORT2		Unpaired t-test		t = 1.15	df1 = 24.00	0.2622	Suppl. 1b
	male	Vehicle1 vs CORT1		Unpaired t-test		t = 1.09	df1 = 21.00	0.2877	Suppl. 1c
		Vehicle2 vs CORT2		Unpaired t-test		t = 0.46	df1 = 24.00	0.6510	Suppl. 1d
**Body Weight loss**	female	Vehicle1 vs CORT1	Relative body weight loss	Unpaired t-test		t = 1.16	df1 = 23.00	0.2565	Suppl. 1e
		Vehicle2 vs CORT2		Unpaired t-test		t = 0.74	df1 = 25.00	0.4637	Suppl. 1f
	male	Vehicle1 vs CORT1		Unpaired t-test		t = 1.05	df1 = 22.00	0.3032	Suppl. 1g
		Vehicle2 vs CORT2		Unpaired t-test		t = 1.61	df1 = 25.00	0.1209	Suppl. 1h
**Drinking volume**	female	Vehicle1 vs CORT1	Relative drinking volume	Unpaired t-test		t = 0.75	df1 = 26.00	0.4595	Suppl. 2a
		Vehicle2 vs CORT2		Unpaired t-test		t = 1.09	df1 = 28.00	0.2841	Suppl. 2b
	male	Vehicle1 vs CORT1		Welch t-test		t = 1.98	df1 = 16.35	0.0653	Suppl. 2c
		Vehicle2 vs CORT2		Welch t-test		t = 2.49	df1 = 17.46	**0.0234**	Suppl. 2d

Posthoc pairwise comparison for bodyweight and Social Interaction test

Experiment	Sex	Comparison		Statistical test	P-value	Figure
		Vehicle1 vs CORT1	Week 1		**0.001**	
		Vehicle1 vs CORT1	Week 2		**0.017**	
	female	Vehicle1 vs CORT1	Week 3	Posthoc pairwise comparison	**0.002**	1b
		Vehicle1 vs CORT1	Week 4		**0.047**	
		Vehicle1 vs CORT1	Week 5-8		P>0.05	
		Vehicle2 vs CORT2	Week1-8	No interaction		1c
Bodyweight		Vehicle1 vs CORT1	Week 3		**0.004**	
		Vehicle1 vs CORT1	Week 4		**0.006**	
		Vehicle1 vs CORT1	Week 6		**0.033**	1d
	male	Vehicle1 vs CORT1	Week 7	Posthoc pairwise comparison	**0.048**	
		Vehicle1 vs CORT1	Week 8		**0.003**	
		Vehicle1 vs CORT1	Week 1,2,5		P>0.05	
		Vehicle2 vs CORT2	Week 1		**P<0.0001**	1e
		Vehicle2 vs CORT2	Week 2-8		P>0.05	

**SI**	female	Vehicle1	Object vs Stranger	Posthoc pairwise comparison	**0.0318**	4b
		CORT1			P>0.05	
		Vehicle2			**0.0139**	4c
		CORT2			P>0.05	
	male	Vehicle1			P>0.05	4d
		CORT1			P>0.05	
		Vehicle2			**P<0.0001**	4e
		**CORT2**			**0.0040**	
