# Supplementary figures and images for: Effect of Chronic Corticosterone Treatment on Depression-Like Behavior and Sociability in Female and Male C57BL/6N Mice
Source: Cells. 2019 Sep 1;8(9):1018. doi: 10.3390/cells8091018 (PMC6770122; doi:10.3390/cells8091018)

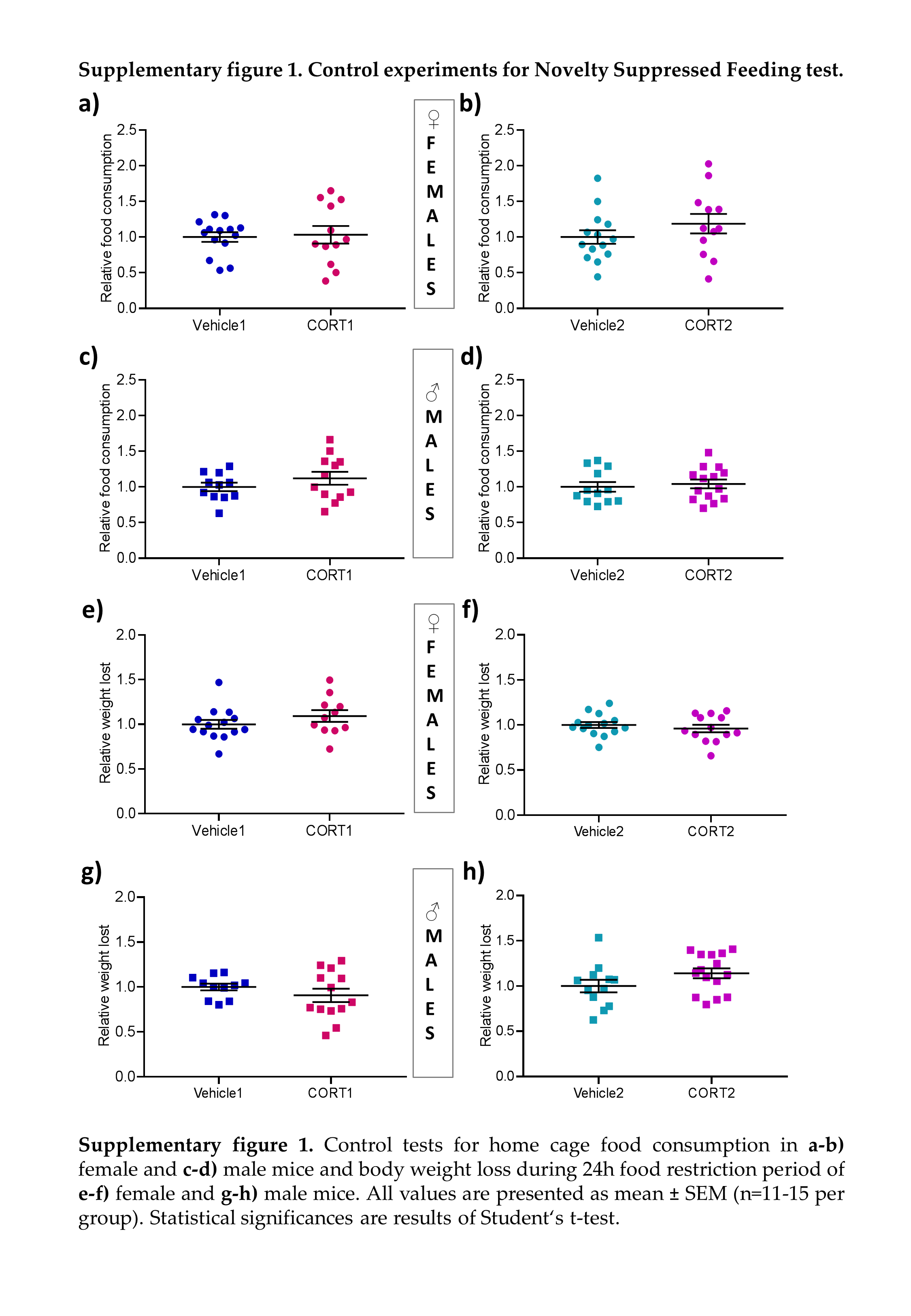

Supplement: Supplementary file 1 [file cells-08-01018-s001.zip › cells-560950-supplementary/SUPPL/Supplimentary Figure 1.tif]

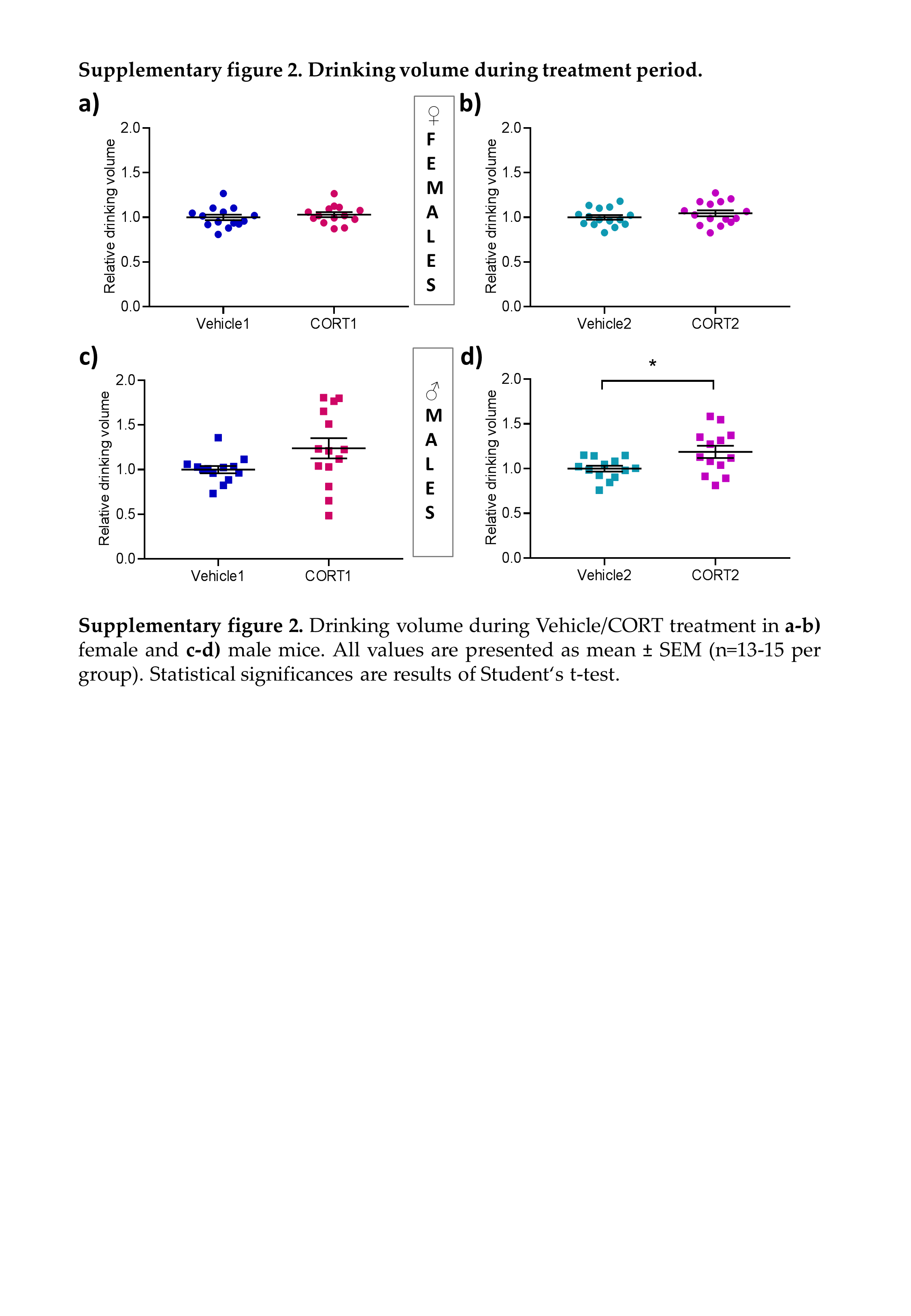

Supplement: Supplementary file 1 [file cells-08-01018-s001.zip › cells-560950-supplementary/SUPPL/Supplimentary Figure 2.tif]
